# The significant influence of residual feed intake on flavor precursors and biomolecules in slow-growing Korat chicken meat

**DOI:** 10.5713/ab.20.0736

**Published:** 2021-02-15

**Authors:** Chotima Poompramun, Wittawat Molee, Kanjana Thumanu, Amonrat Molee

**Affiliations:** 1 School of Animal Technology and Innovation, Institute of Agricultural Technology, Suranaree University of Technology, Nakhon Ratchasima, 30000, Thailand; 2 Synchrotron Light Research Institute (Public Organization), Nakhon Ratchasima, 30000, Thailand

**Keywords:** Biomolecules, Flavor Precursor, Physicochemical Properties, Residual Feed Intake, Slow-Growing Chicken

## Abstract

**Objective:**

This study investigated the association between feed efficiency, physicochemical properties, flavor precursors and biomolecules in the thigh meat of Korat (KR) chickens.

**Methods:**

The feed intake and body weight of individual male KR chickens were recorded from 1 to 10 weeks old to calculate the individual residual feed intake (RFI) of 75 birds. At 10 weeks of age, chickens with the 10 highest (HRFI) and lowest RFI (LRFI) were slaughtered to provide thigh meat samples. The physicochemical properties (ultimate pH, water holding capacity [WHC], drip loss) and flavor precursors (guanosine monophosphate, inosine monophosphate (IMP), adenosine monophosphate and inosine) were analyzed conventionally, and Fourier transform infrared spectroscopy was used to identify the composition of biomolecules (lipids, ester lipids, amide I, amide II, amide III, and carbohydrates) and the secondary structure of the proteins. A group t-test was used to determine significant differences between mean values and principal component analysis to classify thigh meat samples into LRFI and HRFI KR chickens.

**Results:**

The physicochemical properties of thigh meat samples from LRFI and HRFI KR chickens were not significantly different but the IMP content, ratios of lipid, lipid ester, protein (amide I, amide II) were significantly different (p<0.05). The correlation loading results showed that the LRFI group was correlated with high ratios of lipids, lipid esters, collagen content (amide III) and beta sheet protein (rg loading >0.5) while the HRFI group was positively correlated with protein (amide I, amide II), alpha helix protein, IMP content, carbohydrate, ultimate pH and WHC (rg loading >0.5).

**Conclusion:**

The thigh meat from chickens with different RFI differed in physiochemical properties affecting meat texture, and in the contents of flavor precursors and biomolecules affecting the nutritional value of meat. This information can help animal breeders to make genetic improvements by taking more account of traits related to RFI.

## INTRODUCTION

Consumers consider that meat from slow-growing chickens has a better flavor and texture compared with commercial chicken breeds, thus leading to an increased demand for slow-growing chicken breeds [1]. The Korat chicken (KR), a slow-growing line that is a crossbreed between the indigenous Thai Leung Hang Khao and the Suranaree University of Technology (SUT) synthetic line, produces meat with a good flavor and low purine content [2]. However, the KR chicken has the disadvantage of low feed efficiency leading to high costs of production, and thus provides challenges to the poultry feed industry in managing increased costs [3] because of reductions in areas available for feed production and the effects of climate change [4]. Therefore, optimizing feed efficiency and meat quality is an important goal for poultry producers needing to increase profits while being environmentally responsible.

Residual feed intake (RFI), an alternative measure of feed efficiency, is defined as the difference between the measured feed intake and the expected feed intake based on a multiple regression equation involving production and body weight maintenance requirements [5]. Based on the RFI concept, any reduction in feed intake to produce a unit of chicken product could minimize feed costs and thus maximize overall profitability for the poultry industry. Selecting a breed for decreased RFI is related to reducing the feed intake [6] without sacrificing growth performance and production.

Although improving RFI has no effect on the performance of the animal, it is possible that genetic improvements for RFI may adversely affect the physicochemical properties, flavor precursors and biochemical compound in the meat. Liu et al [7] and Lee et al [8] have revealed that high and low RFI chickens were differentially regulated regarding nutrient digestion, protein synthesis, lipid metabolism, molecular transport of cellular molecules and absorption pathways. Abasht et al [9] have also shown that high and low feed efficiency chickens exhibited different regulation of metabolism pathways such as nucleotide sugar biosynthesis, glycogen metabolism and lipid uptake and transport. This suggests that improving feed efficiency as shown by RFI might be associated with the accumulation of biochemical compounds and flavor precursor in the muscle. Unfortunately, few studies have attempted to clarify or confirm the association between RFI and these meat quality parameters in slow-growing chicken except for that by Wen et al [10], who reported that RFI was not significantly correlated with the subcutaneous fat thickness or the intramuscular fat content of either the breast or thigh meat of slow-growing chickens. Therefore, the present study hypothesized that chickens with different RFI should have different meat qualities, in terms of their physicochemical properties, flavor precursors and biochemical compounds.

Regarding flavor precursors, Jayasena et al [11] reported that unsaturated fatty acids, free amino acids, and nucleotides content (i.e., inosine-5’-monophosphate [IMP], adenosine-5’-monophosphate [AMP], inosine) in meat can lead to characteristic meat flavors particularly in chicken and pork. The present study, therefore, used the contents of the nucleotides, IMP, AMP, and inosine as representative flavor precursors.

Fourier transform infrared (FTIR) is a technique used for detecting molecular vibrations which generate narrow and sharp peaks in the mid-infrared region of wavenumbers between 3,000 and 900 cm^−1^. Each type of biomolecule has a distinct and characteristic functional group (Table 1). FTIR spectroscopy is a highly sensitive technique that can detect very small changes in the content of the molecule [12]. The technique is also suitable for analyzing biological samples because it is rapid and non-invasive compared with traditional methods with their limitations of being more time-consuming, destructive, and expensive, and requiring experienced operators [13,14]. Previous studies have used FTIR to investigate or monitor changes in the biochemical molecules in meat to determine any changes in the secondary structures of beef protein at different temperatures [15,16] and to measure the differences in water properties and the structural organization of proteins between normal-pH pork and high-pH pork [17]. Therefore, FTIR was used in the present study to reveal the changes in the key biomolecules of chicken muscle associated with different levels of feed efficiency which traditional methods would be unable to explain.

This study focused first on thigh meat because the thigh and drumstick are preferred in East Asia, Mexico, India, Russia, and Morocco [18]. However, no information was available regarding feed efficiency and meat quality for the thigh of slow-growing chickens. Therefore, information on optimizing feed efficiency and meat quality in thigh meat is important for improving feed efficiency without degrading meat quality.

The question arises on how feed efficiency is associated with RFI and physicochemical properties, flavor precursors and biochemical compound. The answer will provide a clearer direction for designing breeding programs for improving the feed efficiency of the chicken without any negative consequences on meat quality. This study therefore aims to investigate the associations between RFI and meat quality as measured by the physicochemical properties, and the contents of flavor precursors and biomolecules in the thigh meat of KR chickens.

## MATERIALS AND METHODS

### Ethical approval

The experiment was conducted following the code of ethics for animal experimentation with prior approval by the Ethics Committee on Animal Use of the Suranaree University of Technology, Nakhon Ratchasima, Thailand (document ID - U1-02631-2559).

### Experimental animals

The slow-growing KR chickens were produced from Leung Hang Khao sires and SUT dams. At hatch, the chickens were sexed by the vent sexing method with 75 male chicks being used for this experiment. The chicks were wing-banded, vaccinated against Marek’s disease then individually weighed. They were brooded in individual cages from 1 day of age and raised under the same environmental and nutritional conditions. All birds were individually fed *ad libitum* using a starter diet (21% protein), grower diet (19% protein), and finisher diet (17% protein) at 0 to 3, 4 to 6, and 7 to 10 weeks of age, respectively. The individual body weight and feed intake of the KR chickens were recorded weekly from 1 week until 10 weeks of age for RFI calculation as described by N’Dri et al [19]. The formula for RFI calculation is shown below:

$$RFIi=Total\;feed\;intake\;for\;{week}_{i}-(b_{0}+b_{1}{MMW}_{i}+b_{2}{BWG}_{i})$$

where: *BWG_i_* is the body weight gain (g) during *week_i_* and *MMW_i_* the metabolic weight estimated from mean body weight at *week_i_*, i.e., ${\left(\frac{Initial\;body\;weight+Final\;body\;weight}{2}\right)}^{0.75}$, *b*_0_ is the intercept, and *b*_1_ and *b*_2_ are partial regression coefficients.

The cumulative RFI from hatch to week_j_ was calculated as:

$$RFIj=\Sigma_{i=1}^{J}RFIi$$

At 10 weeks of age, the chickens were divided into 2 groups: the 10 chickens with the highest RFI (HRFI) and the 10 chickens with the lowest RFI (LRFI). They were then slaughtered by stunning with electricity, bled, scalded at 60°C, de-feathered and eviscerated. The carcasses were washed then chilled at 4°C. Part of the thigh meat was used to measure meat quality.

### Meat sample collection and preparation

The thigh meat samples from the chickens were vacuum-packed individually in plastic bags then placed in a chill room at 4°C for 24 h. The 75 individual thighs were chopped up then spread out in aluminum foil boxes (3×5 cm). The samples were frozen at −80°C for 24 h then freeze dried in a freeze-dryer (Alpha 2–4 LSC plus, Martin Christ Gefriertrocknungsanlagen GmbH, Osterode am Harz, Germany) operating at −80°C and 1.65 Pa for 24 h. The individual samples of freeze-dried thigh meat were ground into powder then stored in a desiccator at 37°C before FTIR spectroscopic analysis.

### Physicochemical properties

#### Ultimate pH measurement

The ultimate pH values of the thigh meat were determined 24 h after slaughter by inserting a pH-electrode (pHCore-kit, Sartorius Lab Instruments GmbH, Goettingen, Germany) into the core of the muscle. Before making the measurements, the pH-meter was calibrated using standardized buffers (pH 4.01 and 7.00) at room temperature.

#### Drip loss measurement

The drip was collected from individual thigh samples weighing approximately 4 to 5 g, which were wrapped in absorbent pads, placed in polyethylene bags, stored for 24 h at 4°C then weighed. The percentage drip loss was calculated as follows:

$$Drip\;loss=[({\text{w}eight}_{before\;storage}-{\text{w}eight}_{after\;storage})\times 100]/{\text{w}eight}_{before\;storage}$$

#### Water-holding capacity measurement

The water-holding capacity (WHC) of the individual thigh muscles was measured as described by Sakata et al [20]. A 5-g sample was weighed, placed on a nylon net then wrapped in 3 layers of filter paper (No. 4, Whatman International Ltd, Maidstone, UK). The wrapped sample was centrifuged at 3,000×g for 20 min (Thermo Fisher Scientific, Langenselbold, Germany). The WHC was calculated as follows:

$$WHC\;(\%)=[(meat\;{weight}_{after\;centrifugation})/(\text{m}eat\;{weight}_{before\;centrifugation}\times 100]$$

### Measurement of adenosine triphosphate-related compounds

The nucleotides content of the individual thigh muscles was measured as described by Jayasena et al [21]. A 5-g sample was weighed, mixed with 30 mL of 0.75 M perchloric acid, homogenized for 30 s, centrifuged at 2,000×g (Thermo Fisher Scientific, Germany) at 4°C for 5 min to extract the nucleic acids then filtered through a filter paper (No.1, Whatman International Ltd., UK). The filtrate (5 mL) was analyzed using a high-performance liquid chromatography (HP 1260, Agilent Technologies, Santa Clara, CA, USA). The peaks of the individual nucleotides were identified using the retention times of standards of hypoxanthine, inosine, IMP, and AMP (Sigma-Aldrich Co., St. Louis, MO, USA), and the concentrations were calculated using the area under each peak.

### Fourier transform infrared spectroscopy

#### Raw spectra

Changes in the concentration profiles of biomolecules in the meat samples were collected using attenuated total reflectance (ATR)-FTIR spectroscopy using a single reflection ATR sampling module with a deuterated triglycine sulfate detector over the measurement range from 4,000 to 400 cm^−1^ with a spectral resolution of 4 cm^−1^ with 64 scans co-added (Bruker Optics Ltd, Ettlingen, Germany). OPUS software (version 7.2, Bruker Optics Ltd, Germany) was used to acquire the spectra and to control the instrument. The infrared spectra were analyzed to extract information on interesting components, including relative quantification and secondary structures.

#### Data analysis for Fourier transform infrared spectra

Based on the extreme values of LRFI and HRFI at 10 weeks, samples from the two groups, 10 extreme LRFI and 10 extreme HRFI birds, were selected for investigating changes in the content of the biomolecules. The raw spectra from each of these groups consisting of 150 spectra (15 spectra/sample) were converted to the 2nd derivative using 13 smoothing points, reduced from 15 spectra to 3 spectra by averaging over the technical replicates then vector normalized to take account of the effects of differing sample thickness using the Savitzky-Golay method in the Unscrambler X software (version 10.1, Camo Analytics, Oslo, Norway).

There are several methods to normalize spectra - min-max normalization, offline normalization, and vector normalization. For the present study, we selected vector normalization, the optimum procedure to consider the effect of the thickness of a biological sample. All spectra from the samples were preprocessed using vector normalization and the Unscrambler X program to reduce the effect of sample thickness. The normalization method, known as extended multiplicative scatter correction, uses prior knowledge such as extra parameters that can account for the physical or chemical phenomena that can affect the spectra particularly for biological samples [22].

#### Percentage of integrated area calculation

The spectra were preprocessed using the second derivative which can help resolve nearby peaks and sharpen spectral features. To focus on changes in the spectral regions from 3,000 to 2,800 cm^−1^ and 1,800 to 900 cm^−1^ in the main spectrum, we used the second derivative and normalized the spectra to obtain the integral area of each functional group.

To determine the ratio of the biomolecules, the processing spectra from the 10 extreme LRFI and 10 extreme HRFI birds were integrated in several regions: 3,000 to 2,800 cm^−1^ (CH stretching from lipid); 1,740 cm^−1^ (C=O ester from lipid); 1,700 to 1,600 cm^−1^ (amide I); 1,600 to 1,500 cm^−1^ (amide II); 1,338 cm^−1^ (amide III); and 1,250 to 900 cm^−1^ (carbohydrate and glycogen) using OPUS software (version 7.2, Bruker Optics Ltd, Germany). The peaks in the raw spectra changed sign and became negative peaks with lobes on either side in the second derivative.

#### Curve fitting for the amide I band data

Curve fitting was used to investigate the area of the overlapping peaks of the amide I band in the FTIR spectra using a non-linear least-squares curve-fitting technique based on Gaussian and Lorentzian functions. The fitting parameters (i.e., beta sheet, alpha helix, beta turn, and antiparallel), the peak positions and band shapes were determined from the measured FTIR spectra depending on the type of sample.

### Statistical analysis

#### Significant difference analysis

The group Student’s t-test was used to determine the significance of differences between the mean values of body weight, physicochemical properties, and flavor precursor (nucleotide) contents of the thigh meat from the LRFI and HRFI chickens. The level of significance was defined as alpha = 0.05. SPSS 16.0 statistical software (SPSS Inc., Chicago, IL, USA) was used for the analysis.

#### Multivariate data analysis

A matrix of data of the interactions of the spectra from each cluster of extreme LRFI and HRFI samples, physicochemical properties, flavor precursors, secondary protein structures and biomolecules (spectra intensity from 3,500 to 950 cm^−1^) from FTIR was created then the clustering of the variables was analyzed using principal component analysis (PCA). The sample properties and relationships between variables were identified simultaneously using Bi-plots obtained by calculations from a two-dimensional scatter plot of PCA with the dominant spectral band of the different variables.

The level of correlation between the physicochemical properties, flavor precursors, secondary structures, and biomolecules for each cluster of the extreme LRFI and HRFI samples in the data matrix was calculated using PCA by weighting using a standard deviation of all data then a bi-plot correlation between variables was produced.

## RESULTS AND DISCUSSION

### Meat quality of thigh meat and feed intake accumulation, body weight of LFRI and HFRI KR chickens

The mean values and standard errors of RFI, feed intake accumulation, body weight, physicochemical properties, and flavor precursors of the thigh meat samples of KR chickens are shown in Table 2. The difference between the mean values of feed intake accumulation for the LRFI and HRFI KR chickens was significant (p = 0.00) while the difference in body weight was not significant (p = 0.15). The differences between the mean values of the physicochemical properties (ultimate pH, WHC, drip loss) of thigh meat from the LRFI and HRFI KR chickens were not significant except for the IMP content with a significant difference at p = 0.03.

These results on feed intake and body weight agreed with those of Liu et al [7], who reported that the feed intake of LRFI broiler chickens was significantly lower than that of HRFI broiler chickens, but the body weights were not significantly different. Wen et al [10] studied slow-growing chickens, reporting a significant positive correlation between RFI and total feed intake (rg = 0.62) and a non-significant correlation with body weight. Both studies suggested that different values of RFI could reduce feed intake with no change in weight gain, because the calculation of RFI takes account of the amount of feed intake for individual chickens thus providing a balance between production and the maintenance of body weight [23].

IMP was the only flavor precursor whose content was significantly different between the LFRI and HRFI chickens. In the present study, the results on the physicochemical properties did not align with those when PCA was applied to analyze the correlations using the loading and score plots. This will be explained and discussed in the section on the correlation loading of FTIR spectra with the physicochemical properties and flavor precursors of the thigh meat of LFRI and HFRI KR chickens.

### Determining the ratios of biomolecules and secondary structure proteins in KR chicken thigh meat from the FTIR characteristics

**Figure 1:**
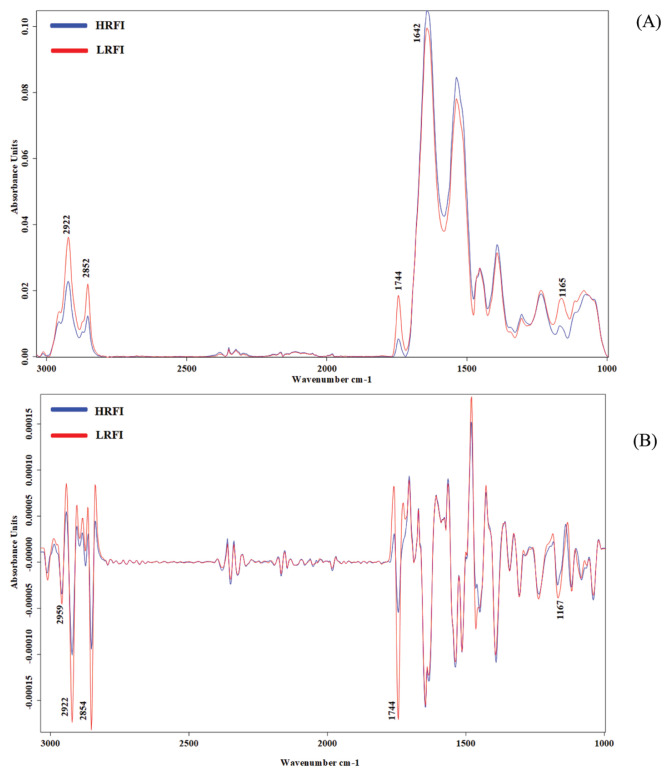
(A) Comparison of average original spectra from thigh meat samples of HRFI and LRFI Korat chickens. (B) Comparison of 2nd derivative spectra from thigh meat samples of HRFI and LRFI Korat chickens. The infrared spectra were detected in the spectral region from 4,000 to 400 cm^−1^, resolution 4 cm^−1^, based on 64 scans. HRFI, high residual feed intake; LRFI, low residual feed intake.

**Figure 2:**
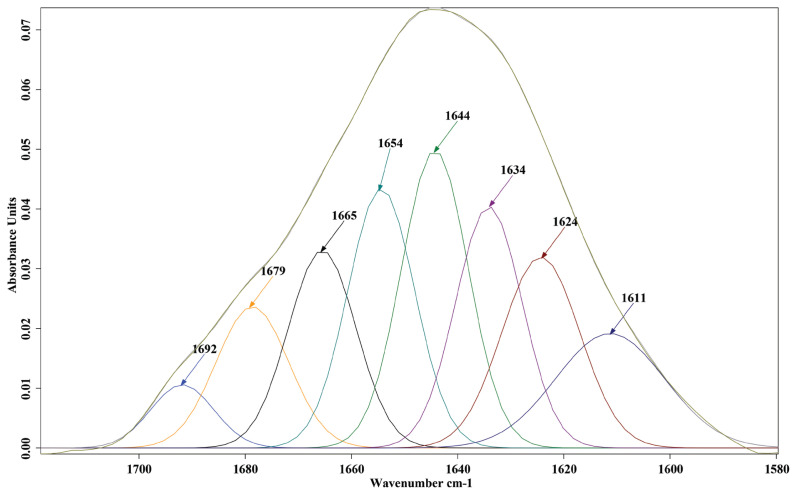
The curve fitting of amide I and secondary structure protein band assignment in thigh meat samples from Korat chickens by residual feed intake.

The average raw spectral features of thigh meat from the LRFI and HRFI KR chickens in the fingerprint region of wave numbers from 3,000 to 900 cm^−1^ are shown in Figure 1A and 1B. The average spectra from the LRFI and HRFI thigh meat samples revealed distinct differences in the peak heights and peak ratios of biomolecules: in the regions 2,922 and 2,854 cm^−1^ representing lipid, 1,744 cm^−1^ representing protein (amide I) and 1,167 cm^−1^ representing carbohydrate and glycogen. The areas under the peaks in these regions were integrated and the percentages of the integrated area for each biomolecule are shown in Table 3. The secondary structures of protein for the amide I band region from 1,700 to 1,600 cm^−1^ were examined by curve fitting as presented in Figure 2. The amide I band revealed that beta sheet can be defined by the regions at 1,611, 1,624 and 1,634 cm^−1^, alpha helix at 1,644, 1,654, and 1,665 cm^−1^, beta turn at 1,679 cm^−1^ and antiparallel at 1,692 cm^−1^. The percentages of each type of secondary structure from curve fitting for LRFI and HRFI chickens are compared in Table 4.

Table 3 shows that the ratios of lipid (shown by FTIR as C-H stretching), lipid ester (>C=O stretching), amide I (C=O stretching) and amide II (C-N stretching + N-H bending coupled out of face) of the thigh meat samples from the LRFI and HRFI KR chickens were significantly different with p-values of 0.00, 0.01, 0.00, and 0.04, respectively. The LRFI chicken samples also exhibited higher ratios of lipids and lipid ester but lower ratios of protein (amide I, amide II) compared with the HRFI chicken samples, while the secondary structure proteins (beta sheet, alpha helix, beta turn and antiparallel) of the HRFI and LRFI chicken samples were not significantly different with p-values of 0.66, 0.76, 0.81, and 0.97, respectively.

A higher fat deposition and lower protein content were detected in the thigh meat samples from LRFI KR chickens, possibly because when an animal receives feed more than its requirements, this source of energy will be converted to fat via lipogenesis then accumulate in the adipose tissues [24]. However, the LRFI chickens consumed feed in significantly lower quantities than the HRFI chickens, thus implying that LRFI chickens utilized energy more efficiently than the HRFI chickens. Therefore, they would need less energy for maintenance than the HRFI chickens, with the excess energy being converted to fat and stored in the body, an interpretation confirmed by reports from Yi et al [25] and Rosa et al [26]. The finding that the thigh meat samples from LRFI KR chickens had a lower protein content than those of the HRFI KR chickens may be explained by reports from Masgrau et al [27] and Yuan et al [28]. These studies reported that the intramuscular deposition of lipids was positively correlated with insulin resistance, so that some intermediates in the insulin signaling pathway were inhibited for the control of protein translation initiation, leading to a lower protein content.

No significant differences were observed in the secondary structure proteins between samples of thigh meat from LRFI and HRFI KR chickens, but these results disagreed with those from the correlation loading. A possible explanation for this will be discussed in the next subtopic.

### Correlation loading of FTIR spectra with the physicochemical properties, flavor precursor (nucleotides) content of the thigh meat from LFRI and HFRI KR chickens

**Figure 3:**
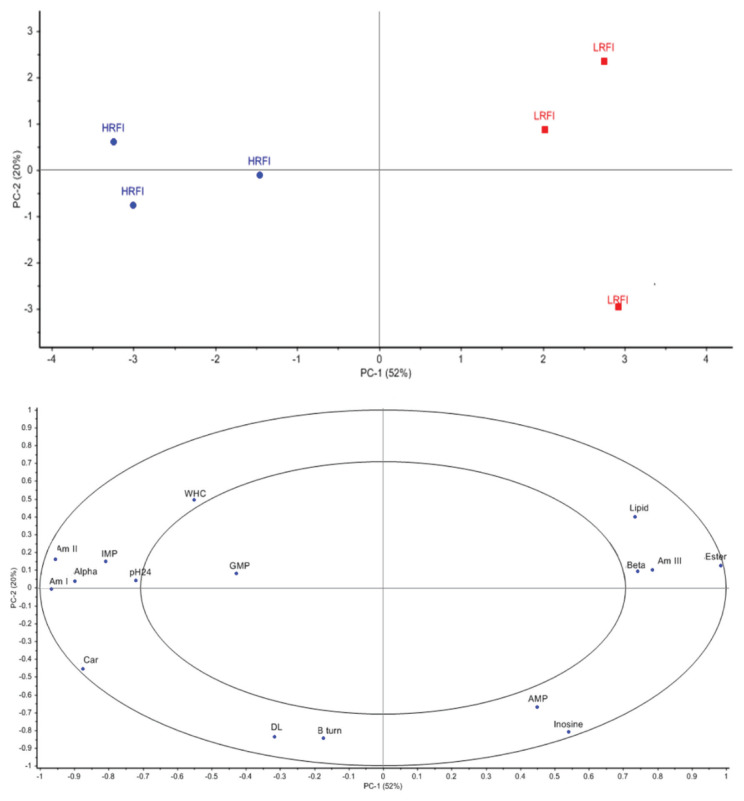
PCA score plot for PC1 versus PC2 from HFRI and LFRI data (upper) and correlation loading plot for PC1 versus PC2 for physicochemical properties, flavor precursors, biomolecules, secondary structure protein (lower). PCA, principal component analysis; PC, principal components; LRFI, low residual feed intake; HRFI, high residual feed intake; pH24, ultimate pH; WHC, water holding capacity; DL, drip loss; GMP, guanosine monophosphate; IMP, inosine monophosphate; AMP, adenosine monophosphate; Am I, amide I; Am II, amide II; Am III, amide III; Ester, ester lipid; Car, carbohydrate; Alpha, alpha helix; Beta, beta sheet; B turn, beta turn.

The present study is the first to combine data from FTIR spectra relating to biomolecules, physicochemical properties and flavor precursors then use PCA for classifying LRFI and HRFI in chickens. The results of a semi-quantitative analysis are shown in Figure 3. The score plots (Figure 3, upper) focusing on PC-1 (horizontal axis) enabled the HRFI and LRFI KR chickens to be differentiated. All variables located in the outer circle region (ultimate pH, WHC, drip loss, IMP, AMP, inosine, lipid, ester bond, amide I, amide II, amide III, beta sheet, alpha helix, and beta turn) showed a significant correlation loading with RFI with a variance greater than 50%. The left-hand side of the loading plot (Figure 3, lower) shows that IMP, amide I (1,700 to 1,600 cm^−1^), amide II (1,600 to 1,500 cm^−1^), alpha helix (1,644, 1,654, and 1,665 cm^−1^), carbohydrate and glycogen (1,250 to 900 cm^−1^), ultimate pH, WHC were positively correlated with HRFI. Although beta turn (1,679 cm^−1^) and drip loss were in the outer circle region, their positions were close to the PC-1 axis meaning that they could not be used for differentiating between the thigh meat samples from HRFI and LRFI KR chickens. However, the right-hand side of the loading plot shows that lipid (3,000 to 2,800 cm^−1^), ester lipid (1,740 cm^−1^), amide III (1,338 cm^−1^) and beta sheet (1,611, 1,624, and 1,634 cm^−1^) were positively correlated with LRFI. The location of AMP and inosine close to the PC-2 axis means that they could not be used for differentiating between the thigh meat samples from HRFI and LRFI KR chickens.

Significant loading correlations were found when PCA was applied, but the results for some variables disagreed with those from the t-test analysis. This is because PCA allows the analysis of datasets containing imprecise measurements by extracting the important information from the data then expressing this information as a set of summarizing indices known as principal components (i.e., PC1, PC2) which can reveal differences in the data matrix [29].

The results from PCA revealed some interesting relationships: the meat samples from HRFI KR chickens were associated with high levels of carbohydrate represented by glycogen. One question is why more glycogen is to be found in the thigh meat of HRFI KR chickens. Glycogen is usually stored in the muscles and liver as an energy reserve [30]. Therefore, the levels of carbohydrate in HRFI KR chicken were significantly higher than in LRFI KR chickens, demonstrating their greater energy reserve. Unfortunately, few studies have explained why HRFI chickens can store more energy than LRFI chicken, except for the studies of Iqbal et al [31] and Van Eerden et al [32], who reported that chickens with a high RFI would be more likely to respond to stress than those with a low RFI. Animals respond to stress by using energy from the breakdown of glycogen. This infers that HRFI KR chickens accumulated more glycogen for use in times of need than LRFI KR chickens.

When animals need energy, glycogen in the muscles is degraded to generate adenosine triphosphate (ATP) [33]. The ATP will then be converted into IMP via adenosine-5-diphosphate, with the degradation of AMP and IMP continuing to produce inosine [34]. This could explain the positive association between HRFI chicken meat and glycogen and IMP, but the other flavor precursors, AMP and guanosine monophosphate (GMP), were shown by PCA to have no association with either LRFI or HRFI. It is possible that storing the meat at 4°C for 24 h before analysis inhibited the processes of AMP or GMP conversion [35].

The association between the secondary proteins, amide I, amide II, and alpha helix, and high protein in HRFI could be explained by insulin resistance control when intramuscular lipids have accumulated as discussed earlier regarding the lower protein content of LRFI KR chickens (Table 3). The high content of alpha helix in the HRFI KR chickens could be explained by the work of da Silva-Buzanello et al [36]. They reported that a high scalding temperature during the chicken slaughtering process could make conformational changes in protein by decreasing the alpha helix and increasing the beta sheet contents and the proportions of beta turn in the breast meat which contains the highest levels of polyunsaturated fatty acids. It could thus be possible that, in the present study, incubating the powdered thigh meat samples at 37°C before the FTIR measurements would not have affected thigh meat samples containing low levels of fat but would have caused lipid oxidation in meat containing high levels of lipid, such as the LRFI thigh meat, leading to conformational changes and a high content of beta sheet.

In the thigh meat from LRFI KR chickens, the association with fat, amide III, led to higher levels of fat deposition because these chickens needed less energy for maintenance leading to an excess of energy, which was then converted into intramuscular fat as mentioned earlier in Table 3. The high collagen levels (amide III) in the thigh meat of LRFI KR chickens could be explained by the reports by Pincu et al [37] and Berria et al [38] who found that high levels of fat deposition in skeletal muscle were strongly associated with insulin resistance. This can result in mitochondrial dysfunction in the composition of the extracellular matrix where changes in the matrix induce greater deposition of collagen in the skeletal muscle.

## CONCLUSION

This study has confirmed that improving feed efficiency by decreasing RFI could significantly reduce feed intake without sacrificing body weight gain. It can also be suggested that KR chickens utilize energy more efficiently, so molecular mechanisms involved with energy utilization should be investigated further to gain a deeper understanding. The results have also strongly signaled that these improvements may adversely affect the physicochemical properties, particularly ultimate pH, and ability to contain water in meat. Further possible consequences of improving RFI are a deterioration in the nutritional values of the meat in terms of its contents of lipids and protein, and the flavor precursor, IMP. Simultaneously, the meat from chickens with low feed efficiency contains more glycogen, so it can be hypothesized that these chickens would be more likely to respond to stress. However, further studies are needed to prove such a hypothesis. Overall, animal breeders would need to take more account of the advantages and disadvantages of this feed efficiency trait when making genetic improvements to chicken breeds.

## Figures and Tables

**Table 1:** Relationship between molecular functional group and biomolecules

Wave number (cm^−1^)	Molecule functional group assignment	Biomolecule	References
2,959, 2,955, 2,938, 2,934, 2,930, 2,921, 2,898, 2,872, 2,870, 2,850	C-H stretching	Fatty acids	[13,14]
1,740	>C=O stretching	Cholesterol esters, Triglyceride esters	[14]
1,695 to 1,675	C=O stretching	Amide I of proteins	[14]
1,655	C=O stretching	Amide I of alpha-helices of proteins	[13,14]
1,637	C=O stretching	Amide I of beta-sheets of proteins	[13,14]
1,550 to 1,520	C-N stretching + N-H bending coupled out of face	Amide II of proteins	[14]
1,310 to 1,240	C-N stretching + N-H bending coupled in of face	Amide III of proteins	[13,14]
1,200 to 900	C-O-C, C-O dominated by ring vibrations of carbohydrates C-O-P, P-O-P	Carbohydrate and glycogen	[13]

**Table 2:** Feed intake, body weight, physicochemical properties, and flavor precursors of thigh meat samples from low and high residual feed intake Korat chickens at 10 weeks of age (mean±standard error)

Parameters	N = 20		p-value

	LRFI	HRFI	
RFI	−558.48±47.22	584.28±58.23	0.00^**^
FI (g)	3,100.15±44.99	4,256.25±63.24	0.00^**^
BW (g)	1,399±41.96	1,508±59.34	0.15
Ultimate pH	6.14±0.10	6.24±0.09	0.45
WHC (%)	81.41±1.19	83.69±1.67	0.28
Drip loss (%)	10.57±1.79	11.83±0.94	0.56
GMP (mg/g)	0.14±0.01	0.15±0.01	0.39
IMP (mg/g)	3.92±0.21	4.72±0.25	0.03^*^
AMP (mg/g)	0.11±0.01	0.10±0.01	0.35
Inosine (mg/g)	0.49±0.06	0.43±0.03	0.32

RFI, residual feed intake; LRFI, low residual feed intake; HRFI, high residual feed intake; FI, feed intake; BW, body weight; WHC, water holding capacity; DL, drip loss; GMP, guanosine monophosphate; IMP, inosine monophosphate; AMP, adenosine monophosphate.

** p<0.001,

* p<0.05.

**Table 3:** Ratio of biomolecules determined by Fourier transform infrared in thigh meat samples from low and high residual feed intake Korat chickens (percentage of integral area±standard errors)

Biomolecule (wavenumber)	% Integral area		p-value

	LRFI	HRFI	
Lipid (3,000 to 2,800 cm^−1^)	25.55±0.65	16.58±0.65	0.00^**^
Ester lipid (1,740 cm^−1^)	10.89±1.06	2.28±1.06	0.01^*^
Amide I (1,700 to 1,600 cm^−1^)	18.96±0.89	27.01±0.89	0.00^**^
Amide II (1,600 to 1,500 cm^−1^)	16.60±1.19	21.60±1.19	0.04^*^
Amide III (1,338 cm^−1^)	0.55±0.35	0.50±0.07	0.68
Carbohydrate (1,250 to 900 cm^−1^)	13.13±0.77	15.17±0.77	0.13

LRFI, low residual feed intake; HRFI, high residual feed intake.

** p<0.001,

* p<0.05.

**Table 4:** Ratio of secondary structure protein in thigh meat samples from low and high residual feed intake Korat chickens (percentage of curve fitting±standard errors)

Secondary protein structure (wavenumber)	% Curve fitting±standard error		p-value

	LRFI	HRFI	
Beta sheet (1,611, 1,624, 1,634 cm^−1^)	36.03±0.54	35.66±0.54	0.66
Alpha helix (1,644, 1,654, 1,665 cm^−1^)	50.77±0.60	51.05±0.60	0.76
Beta turn (1,679 cm^−1^)	9.14±0.28	9.24±0.28	0.81
Antiparallel (1,692 cm^−1^)	4.06±0.18	4.05±0.18	0.97

LRFI, low residual feed intake; HRFI, high residual feed intake.

* p<0.05.
